# A tissue-engineered model of the blood-tumor barrier during metastatic breast cancer

**DOI:** 10.1186/s12987-023-00482-9

**Published:** 2023-11-03

**Authors:** Raleigh M. Linville, Joanna Maressa, Zhaobin Guo, Tracy D. Chung, Alanna Farrell, Ria Jha, Peter C. Searson

**Affiliations:** 1 Institute for Nanobiotechnology, Johns Hopkins University, 100 Croft Hall, 3400 North Charles Street, Baltimore, MD 21218 USA; 2 Department of Biomedical Engineering, Johns Hopkins University, Baltimore, MD USA; 3 Department of Materials Science and Engineering, Johns Hopkins University, Baltimore, MD USA

## Abstract

Metastatic brain cancer has poor prognosis due to challenges in both detection and treatment. One contributor to poor prognosis is the blood–brain barrier (BBB), which severely limits the transport of therapeutic agents to intracranial tumors. During the development of brain metastases from primary breast cancer, the BBB is modified and is termed the ‘blood-tumor barrier’ (BTB). A better understanding of the differences between the BBB and BTB across cancer types and stages may assist in identifying new therapeutic targets. Here, we utilize a tissue-engineered microvessel model with induced pluripotent stem cell (iPSC)-derived brain microvascular endothelial-like cells (iBMECs) and surrounded by human breast metastatic cancer spheroids with brain tropism. We directly compare BBB and BTB in vitro microvessels to unravel both physical and chemical interactions occurring during perivascular cancer growth. We determine the dynamics of vascular co-option by cancer cells, modes of vascular degeneration, and quantify the endothelial barrier to antibody transport. Additionally, using bulk RNA sequencing, ELISA of microvessel perfusates, and related functional assays, we probe early brain endothelial changes in the presence of cancer cells. We find that immune cell adhesion and endothelial turnover are elevated within the metastatic BTB, and that macrophages exert a unique influence on BTB identity. Our model provides a novel three-dimensional system to study mechanisms of cancer-vascular-immune interactions and drug delivery occurring within the BTB.

**Supplementary Information:**

The online version contains supplementary material available at 10.1186/s12987-023-00482-9.

## Introduction

Brain metastases occur in 10–20% of adult cancer patients, with breast cancer representing the second most common primary tumor source [1]. Patients with brain metastases display poor prognosis with short survival and substantial neurological deterioration. The formation of brain metastases is a multi-step process: following intravasation into circulation, tumor cells are arrested by occlusion or adhesion in the cerebrovasculature, extravasate into the perivascular space surrounding the blood–brain barrier (BBB), and proliferate in a metastatic niche [2]. During colonization, metastatic tumor cells create a microenvironment that transforms the BBB into the blood-tumor barrier (BTB) [3].

There are several factors that make studies of the BTB challenging: (1) the scarcity of human tissue (particularly in early stage disease), (2) the use of in vivo rodent models (based on cardiac injection of brain-seeking cancer cell lines) which may not mimic the characteristics of the human BBB, and (3) the lack of physiologically accurate in vitro models [3]. Fabrication of three-dimensional (3D) tissue-engineered models overcomes some of these challenges by using human cells within microenvironments that better mimic the BTB (e.g. shear stress, cell–matrix interactions, cancer-endothelial interactions) [4–7]. While self-organized microvascular network models have enabled detailed studies of tumor cell extravasation [8], these models have not been able to mimic late-stage phenotypes within the metastatic BTB. Direct templating approaches enable co-culture of cancer cells or cancer spheroids proximal to microvessels while enabling live-cell imaging of tumor-vessel interactions [9–12]; however, to date this approach has not been used to study the BTB phenotype.

To visualize BTB interactions, we created a model by combining single cell suspensions or spheroids of human metastatic breast cancer cells displaying brain tropism with tissue-engineered microvessels formed from induced pluripotent stem cell (iPSC)-derived brain microvascular endothelial-like cells (iBMECs). Our model decouples the complex cell–cell interactions occurring within the metastatic niche between cancer cells, BMECs, perivascular cells, and immune cells. While the influence of cell types (i.e. astrocytes and pericytes) on BTB properties has been studied in animal models [13, 14], the direct effects of cancer cells on brain endothelium in the absence of supporting cells has not been explored. To characterize how cancer cells transform the BBB, we first constructed models with either single cells or cancer spheroids embedded in a hydrogel matrix surrounding a cylindrical 150 μm diameter microvessel. We found that single metastatic cancer cells displayed heterogenous survival and limited growth, while cancer spheroids displayed stable growth over 6 days. Using the spheroid model, we: (1) assessed microenvironmental regulation of cancer growth within in vitro metastatic lesions, (2) explored the dynamics of vascular co-option and mosaic vessel formation by cancer cells, (3) determined the dynamics of BTB permeability to antibodies, (4) assessed changes in gene expression and function of the BBB and BTB endothelium, and (5) characterized the role of macrophage co-culture on BTB phenotype. Our tissue-engineered model provides new insight into changes in BTB phenotype during metastatic breast cancer, which may motivate new therapeutic approaches.

## Materials and methods

### Cell culture

The human JIMT-1-BR cell line (HER2 + , ER/PR −) was developed in the Steeg lab (NCI, Bethesda, MD) [15]. Briefly, JIMT-1-BR cells were established by three passages in vivo from intracardiac injection of cancer cells from a HER2 + Trastuzumab-resistant breast cancer patient [15, 16]. JIMT-1-BR cells were cultured in basal cancer medium: DMEM high glucose medium supplemented with 2 mM L-glutamine (ThermoFisher), 10% fetal bovine serum (Sigma), and 1% pen-strep (ThermoFisher). Cells were routinely passed using 0.25% trypsin EDTA (ThermoFisher) between passage 8 and 12, at a ratio of 1:5 on tissue-culture treated six-well plates. To form cell spheroids, 300,000 cells were passed into a well of an ultra-low attachment six-well plate (Corning) and harvested for incorporation into devices 3 days after passaging.

Brain microvascular endothelial-like cells (iBMECs) were differentiated and then cryopreserved following published protocols [17–19]. WTC iPSCs with red fluorescence protein (RFP)-labeled plasma membrane (AICS-0054 cl.91; Allen Cell Institute) were used for all experiments [20]. Cryopreserved iBMECs were freshly thawed in brain microvessel growth media: human endothelial cell serum-free media (Life Technologies) supplemented with 1% human platelet poor plasma-derived serum (Sigma), 2 ng mL^−1^ bFGF (R&D Systems), and 10 μM all-trans retinoic acid (Sigma), 1% penicillin–streptomycin, and 10 μM ROCK inhibitor Y27632 (ATCC). Cells were cultured on tissue-culture treated plates coated overnight with 50 μg mL^−1^ human placental collagen IV (Sigma) and 25 μg mL^−1^ fibronectin from human plasma (Sigma). After one-hour, medium was replaced with fresh medium, and Accutase (ThermoFisher) was used to collect adherent and viable cells.

### Blood-tumor barrier model

A tissue-engineered microvessel model of the human BBB was fabricated similar to previously reported [18]. While previous work utilized a 7 mg mL^−1^ collagen hydrogel cross-linked with genipin, here, a composite extracellular matrix (ECM) comprised of 6 mg mL^−1^ neutralized rat tail type I collagen (Corning) and 1.5 mg mL^−1^ Matrigel (Corning) (Col-Mg hydrogel) was used for all studies. This hydrogel composition avoids the use of genipin which is toxic to cells and hence cannot be used in studies where cells are seeded in the hydrogel matrix. In addition, this composition enabled robust growth of cancer spheroids (data not shown). Single cells or cancer spheroids were embedded around microvessels at ~ 150,000 cells mL^−1^ to form the BTB model. Col-Mg hydrogel was neutralized on ice, introduced into the PDMS housing around a 150 μm diameter super-elastic nitinol wire, and then gelled for 20 min at 37 °C. To prevent delamination of the collagen gel, 2% agarose was added to both sides of the hydrogel. Gelled devices were kept on ice for 3–6 h to remove air bubbles in the hydrogels before removing the wire and incubating overnight at 37 °C in basal cancer medium to equilibrate cancer cells prior to microvessel formation. iBMECs were resuspended to 1 × 10^7^ mL^−1^ in brain microvessel growth media and incubated under static conditions for 30 min to promote cell adhesion within microchannels. Afterwards, microchannels were perfused with brain microvessel growth media under a shear stress (τ) of ~ 2 dyne cm^−2^ achieved using gravity-driven flow reservoirs as previously reported [18]. After 24 h, the medium was switched to brain microvessel maintenance medium: human endothelial cell serum-free medium supplemented with 1% human platelet poor plasma-derived serum and 1% penicillin–streptomycin.

### Microscopy and image analysis

Phase contrast and epifluorescence images were collected on a Nikon TiE microscope (Nikon Instruments Inc.) with illumination provided by an X-Cite 120LEDBoost (Excelitas Technologies) at 10 × magnification on day 0 (immediately after seeding of iBMECs), and days 2, 4, and 6. Circular ROIs were used to track changes in spheroid area, fluorescence intensity, and distance from the vessel in ImageJ. From phase contrast and epifluorescence images, the frequency of two mechanisms of vascular interactions were quantified: mosaic vessel formation and vascular co-option (cancer cell migration along vessels). Vascular co-option was subdivided according to whether cell migration occurred before or after BTB degeneration. In most figures, the RFP-labeled iBMECs are pseudo-colored magenta for color blind compatibility.

### Microvessel permeability

Two compounds were used to quantify BBB permeability: (1) goat anti-rabbit IgG conjugated to Cascade Blue and (2) human anti-HER2 IgG conjugated to Alexa Fluor^®^ 647. The anti-rabbit IgG is a non-specific IgG (Invitrogen #C2764). The HER2 antibody is a research grade biosimilar for Trastuzumab (R&D #FAB9589R) which mimics the size and specificity, but not the clinical efficacy of Trastuzumab. 80 μg mL^−1^ of non-specific IgG and 20 μg mL^−1^ Trastuzumab biosimilar was added to brain microvessel maintenance medium and then perfused through microvessels for 30 min. We confirmed that antibody exposure (matching concentrations used in microvessels) for 24 h in 2D iBMEC monolayers does not alone alter barrier function of iBMECs (*p* = 0.373, unpaired t-test across *n* = 3 microvessels). Fluorescence images of each conjugated compound, iBMECs, and cancer cells, were acquired every five minutes at 10 × magnification. Images were collected as ten adjacent frames along the microvessel axis, corresponding to a total image area of 8.18 mm × 0.67 mm. ImageJ was used to reconstruct fluorescence intensity profiles over 10 min of background imaging (3 frames) and 30 min of antibody perfusion (7 frames). Permeability (P) was calculated from (d/4)(1/ΔI)(dI/dt)_0_, where d is the microvessel diameter, ΔI is the increase in fluorescence intensity due to luminal filling, and (dI/dt)_0_ is the rate of fluorescence intensity increase [18, 21]. To reduce artifacts due to interstitial flow of antibodies from the inlet and outlet ports, the permeability was calculated across 30 min of perfusion and reported as the mean value of five adjacent frames with the lowest permeability. Focal leaks of solutes were manually counted along the length of microvessels and are reported as a density relative to microvessel length (# cm^−1^). At high densities of focal leaks, adjacent leaks cannot be reasonably distinguished, and we assumed there was one focal leak per 10 × image frame.

### Quantifying solute accumulation

Following perfusion of the fluorescently-labeled molecules on day 2, no fluorescence was detected in the endothelial cells or in the surrounding matrix in BBB or BTB microvessels (i.e. no measurable permeability). Therefore, we utilized the fluorescence on day 4 (prior to the addition of dye for the permeability assay) to measure solute accumulation in three regions of interest (ROIs): the endothelium, cancer spheroids, and extracellular matrix. Solute accumulation was quantified as the ratio of fluorescence on day 4 to day 2, where values greater than one indicate accumulation. No accumulation was detected in the ECM during the 48 h of media perfusion between days 2 and 4, indicating complete fluorophore washout from hydrogels. All ROIs had an area of 2,700 µm^2^ that only contained elements of the individual environment of interest (i.e., no ROIs with spheroids and ECM).

### Endothelial cell proliferation and cell loss analysis

Phase contrast time-lapse images were captured simultaneously with fluorescence images during permeability measurements at the microvessel polar planes. In the phase contrast time-lapse images mitosis and cell loss events were identified by the emergence of daughter cells or collapse of a cell nucleus, respectively. Rates of cellular events were normalized to % h^−1^, where turnover is calculated as the difference between the rates of mitosis and cell loss.

### Immune cell and cancer cell adhesion assays

THP-1 cells (ATCC^®^ TIB-202^™^) were grown in suspension with RPMI-1640 Medium (Sigma) supplemented with 10% fetal bovine serum (Sigma) and 1% penicillin–streptomycin. THP-1 s are a human monocytic cell line derived from a patient with leukemia [22]. Singularized THP-1 s or JIMT-1-BR cancer cells were fluorescently labeled with 1 µM CellTracker^™^ Deep Red Dye (Invitrogen) for 15 min, and then resuspended at 1 × 10^6^ cells mL^−1^ in basal media. THP-1 s or cancer cells were perfused through microvessels under low shear stress (~ 0.2 dyne cm^−2^) for 10 min, before washout using high shear stress (~ 2 dyne cm^−2^) and manual counting of adhered cells.

### Confocal imaging and immunofluorescence

40 × confocal images were obtained using a swept field confocal microscope system (Prairie Technologies) with illumination provided by an MLC 400 monolithic laser combiner (Keysight Technologies) to further visualize tumor-vessel interactions. Immunofluorescence staining was performed within the microvessel device to visualize protein expression. Microvessels were washed with PBS for 5 min, fixed with 4% paraformaldehyde (Sigma) for 15 min, permeabilized with 0.1% Triton X-100 (Sigma) for 30 min, and blocked with 10% goat serum overnight at 4 ˚C. Nuclei were stained using 1:1000 DAPI solution (Invitrogen). Primary antibodies used were: rabbit anti-human PRSS3 (LSBio, #B13831) at a 1:25 dilution, and mouse anti-human ICAM1 (Abcam, #AB2213) at a 1:50 dilution. Secondary antibodies used were: goat anti-rabbit Alexa Fluor 488 (Invitrogen, #A11008), and goat anti-mouse Alexa Fluor 647 (Invitrogen, #A21235), both at a 1:200 dilution. Semi-quantitative analysis of protein expression from mean intensity projections of confocal z-stacks was conducted in ImageJ by relative fluorescence intensity of targets versus DAPI signal, and then normalized to a value of one for BBB microvessels.

### Transendothelial electrical resistance (TEER) measurements

iBMECs were seeded at a density of 1 × 10^6^ cm^−2^ onto Transwells that were treated overnight with 50 μg mL^−1^ human placental collagen IV (Sigma) and 25 μg mL^−1^ fibronectin from human plasma (Sigma). Transendothelial electrical resistance (TEER) values (Ω cm^2^) were recorded using an EndOhm (World Precision Instruments), as previously reported [23]. All measurements were performed on 6.5 mm Transwells with a 0.4 μm pore polyester membrane insert (Corning). TEER values for Transwells with no cells were subtracted from the measured values, and were then normalized to the membrane area. The effects of antibodies, cancer-conditioned medium, and co-cultured cancer cells on TEER were tested (Additional file 1: Fig. S5). The apical chambers of Transwells were exposed to identical concentrations of the fluorescently-labeled molecules as used in 3D assays for 24 h. Conditioned medium was collected from microvessels as the downstream perfusate and stored at −80 °C. The direct effects of co-cultured cancer cells on TEER were tested by seeding the basolateral chamber of Transwells with ~ 50 cancer spheroids, matching the ratio of iBMECs to spheroids used in 3D models.

### Perfusate analysis

Perfusate was collected from the downstream media reservoir following overnight perfusion and pooled across technical replicates (i.e. individual devices). An enzyme-linked lectin assay (ELLA, ProteinSimple) was used for analyte quantification at The Clinical Research Core Laboratory at Johns Hopkins Bayview campus. Analyte concentration is reported as pg mL^−1^ for BBB microvessels, BTB microvessels, and BTB + macrophage microvessels.

### Bulk RNA sequencing

To assess changes in gene expression, iBMECs in microvessels were isolated following experiments. Briefly, microvessels were washed with cold PBS for 10 min before lysing cells by perfusion with RLT buffer (Qiagen). iBMECs were completely removed from microchannels within one minute, minimizing contributions from cancer or immune cells. RNA isolation was performed using the RNeasy Mini Kit (Qiagen). Following library prep using SMART-Seq v4 (Takara, 634893), sequencing was carried out on an Illumina NovaSeq 6000 platform at the Johns Hopkins Single Cell & Transcriptomics Core with paired end 150 bp reads, generating approximately 20 million paired reads per sample. Alignment to reference genome (GRCh38) and quantification of raw read counts was performed using *Rsubread* (Version 2.0.1) [24]; normalization (rlog transformed), visualization, and differential analysis was performed using *DESeq2* (v1.28.1) [25]. Differentially expressed genes (DEGs) were determined using the Wald test with Benjamini–Hochberg correction. Pathway enrichment analysis was conducted via genome-wide ranked list comparisons using Gene Set Enrichment Analysis (GSEA, v4.1.0) for the Molecular Signatures Database (MSigDB) hallmark gene sets with 1000 permutations and a false discovery rate < 0.25; normalized enrichment score (NES) was calculated by the software [26, 27]. Data are deposited in GEO under accession number GSE214831. Findings are benchmarked to recent work profiling endothelium from human tumor samples posted under accession number GSE159851 and were processed identically as described above.

### Immune cell co-culture

Monocytic THP-1 cells were initially cultured as described above. One day after thawing, cells were treated with 50 ng mL^−1^ phorbol 12-myristate 13-acetate (PMA; Sigma) for 24 h to induce macrophage identity as evident by an adherent phenotype. This treatment induces expression of CD68, CD206, and CD204 indicative of M2-polarized macrophages [28]. Longer term exposure of THP-1 cells to PMA induces substantial cytotoxicity and fails to mimic bacterial responses [29], thus PMA was removed during long term culture. Differentiated cells were singularized using 0.25% Trypsin–EDTA (Gibco) before fluorescently labeling using 1 µM CellTracker^™^ Deep Red Dye (Invitrogen) for 20 min. Labeled macrophages were then suspended at 500,000 cells mL^−1^ in the hydrogel matrix along with cancer spheroids.

### Statistical methodology

Statistical analyses were conducted using Prism (GraphPad ver. 8). All experimental values are reported as mean ± standard deviation (SD). A student’s paired or unpaired t-test (two-tailed with unequal variance) was used for comparison of two groups; an analysis of variance (ANOVA) test was used for comparison of three or more groups with *p*-values multiplicity adjusted using a Tukey test. A one-tailed unpaired t-test was used to validate DEGs by immunofluorescence. Linear regression was conducted using least squares fitting with no constraints, where an F-test was used to determine if linear regression produced a statistically significant non-zero slope. A Gehan-Breslow-Wilcoxon test was used to compare lifespan curves across conditions. For calculations of solute accumulation, a one sample t-test was used to compare fold change in fluorescence values to a hypothetical value of 1.0 (i.e. no accumulation). Differences were considered statistically significant for *p* < 0.05, with the following thresholds: *p < 0.05, **p < 0.01, ***p < 0.001.

## Results and discussion

### Tissue-engineered 3D microvessel model of the blood-tumor barrier

**Fig. 1:**
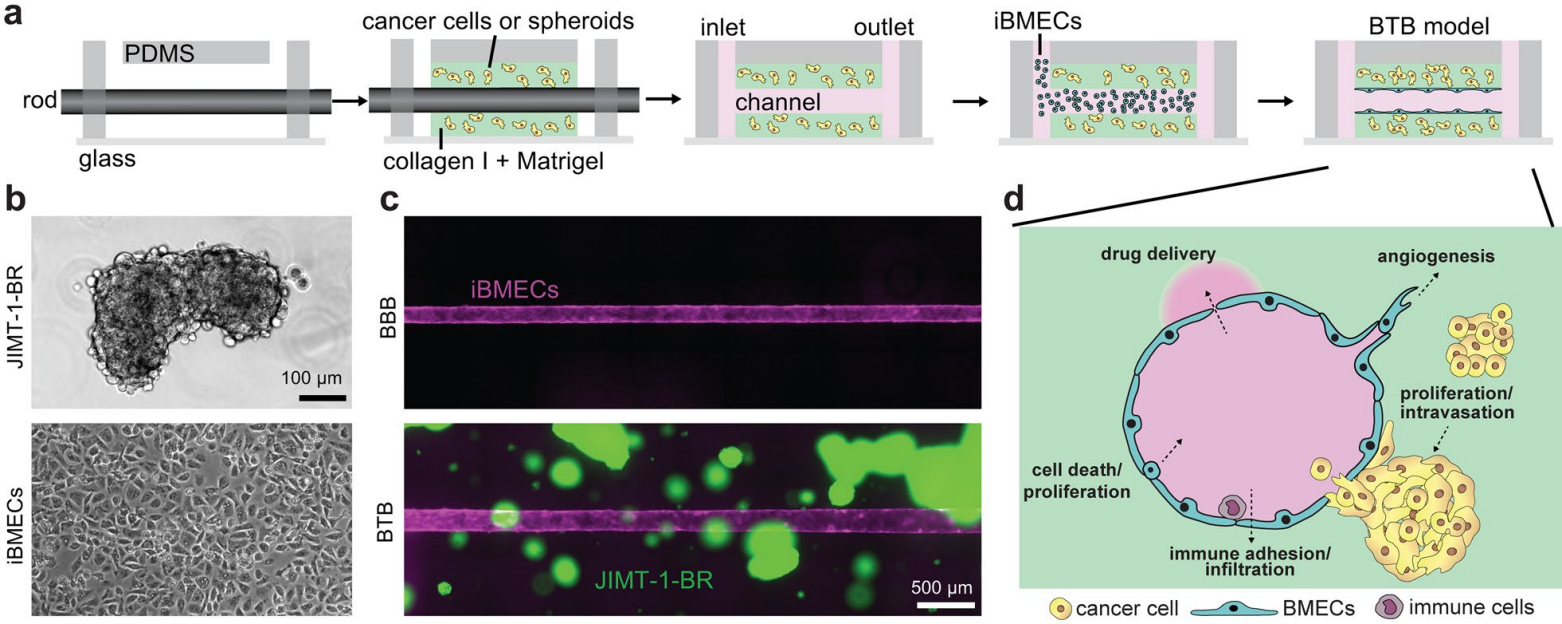
A tissue-engineered model of the blood-tumor barrier during metastatic breast cancer. **a** Schematic of model fabrication. A hydrogel matrix containing tumor cells or spheroids was formed around a template rod. After removal, the channel was seeded with iPSC-derived BMEC-like cells (iBMECs) to form a confluent monolayer. Microvessels were perfused by gravity flow at a shear stress of ~ 2 dyne cm^−2^. **b** Representative phase contrast images of metastatic breast cancer spheroids (JIMT-1-BR) and iBMECs on tissue-cultured treated plates prior to seeding into the BTB model. **c** Representative fluorescence images of the BBB microvessel model (formed by RFP-labeled iBMECs) and BTB model (formed by co-culture with GFP-labeled JIMT-1-BRs) at day 2 after iBMEC seeding. **d** Schematic of model cross-section highlighting key processes occurring within the BTB niche, including cancer cell proliferation, tumor-vessel interactions, vascular proliferation (angiogenesis) or degeneration, regulation of drug delivery, and immune cell interactions

Following the formation of metastatic lesions in the brain, the blood–brain barrier (BBB) displays locally altered structural and functional properties and is termed the blood-tumor barrier (BTB) [3]. To better understand the mechanisms of BTB formation and the interactions between cancer cells and brain microvascular endothelial cells (BMECs), we created a tissue-engineered model with a 150 μm diameter microvessel surrounded by brain metastatic cancer cells within a collagen I and Matrigel matrix [11, 18] (Fig. 1a, b). Both single cells and spheroids were formed using the human JIMT-1-BR cell line which displays brain tropism and is lethal in mice within 3–4 weeks following intracardiac injection due to extensive formation of brain metastases [15]. Spheroids embedded in the model mimicked sizes observed in rodent models of HER2^+^ brain metastases [30]. Microvessels were formed from iPSC-derived BMEC-like cells (iBMECs), which assemble into a confluent endothelium. For direct comparison, we also formed microvessels in the absence of cancer cells mimicking our previously reported model of the BBB [18]. All cell types were fluorescently-labeled to enable live-cell imaging: cancer cells (JIMT-BR-1 s) display stable GFP expression and iBMECs with RFP-labeled plasma membrane (Fig. 1c). To characterize the phenotype of the BTB over the course of 6 days, we utilized phase contrast and fluorescence imaging, functional assays, and analysis of gene and protein expression (Fig. 1d).

### Microenvironmental regulation of cancer cell growth

**Fig. 2:**
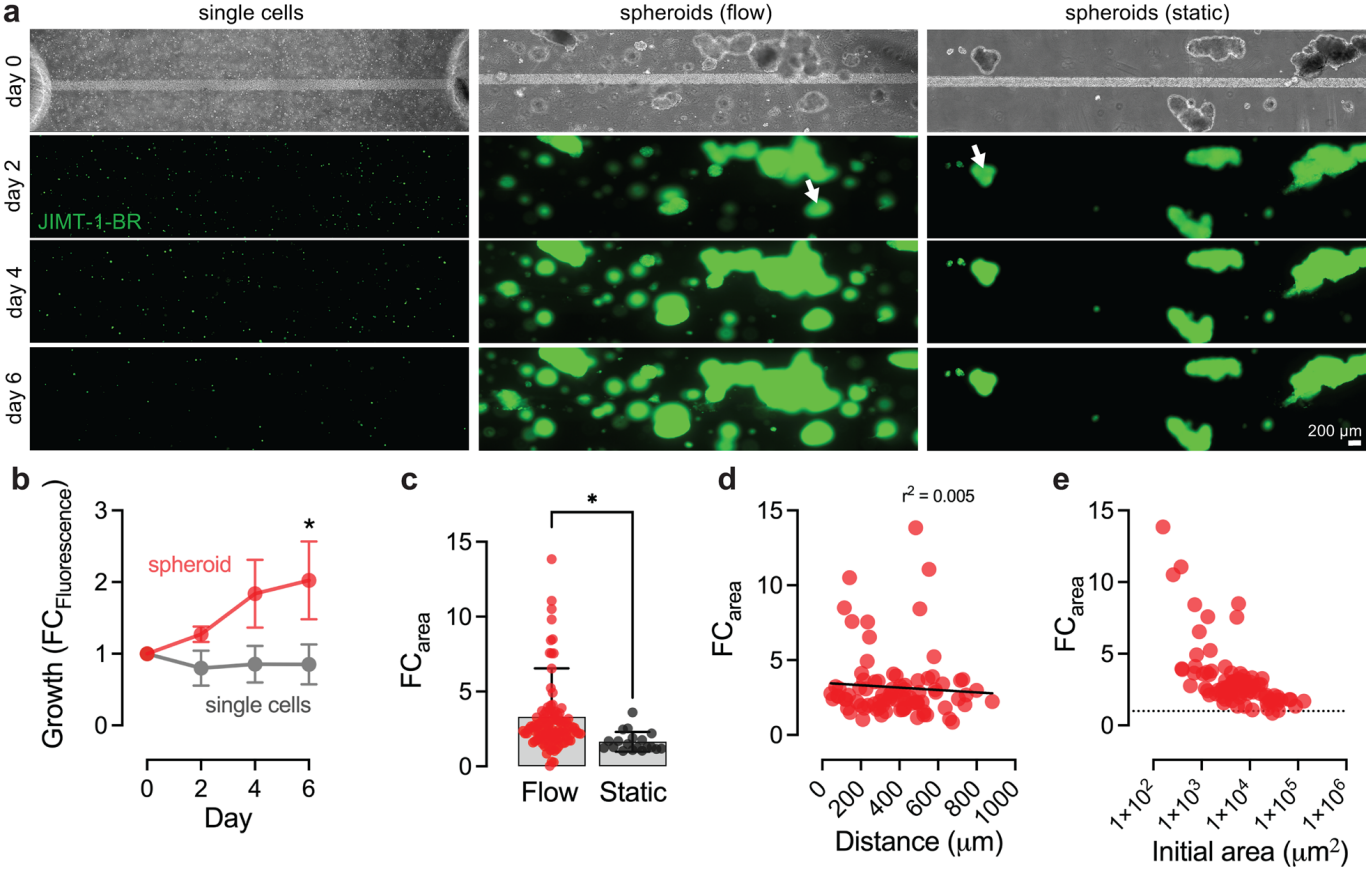
Dynamics of cancer growth within the BTB model. **a** Representative time course images of single cells, spheroids, and spheroids under static conditions in the BTB model. Day 0 images shows phase contrast, while other timepoints show JIMT-1-BRs (green). Tumor cells were seeded at an average density of 150,000 cell mL^−1^. White arrows show similarly sized spheroids at day 0 that grow only under flow conditions. **b** Spheroids showed a twofold increase in fluorescence over six days. Single cells displayed a small decrease in overall fluorescence over six days with both cell loss and proliferation. Data collected across *n* = 3 devices formed using single cells and *n* = 6 devices formed using spheroids. **c** Microvessel perfusion increased growth of cancer spheroids. Spheroid size is reported as the projected area of all spheroids in maximum intensity projections. Data collected across *n* = 85 spheroids under flow (11 independent devices) and *n* = 18 spheroids cultured under static condition (5 independent devices). **d**–**e** Relationship of spheroid growth rate to distance from microvessel and initial spheroid size. The growth rate was determined from the difference in projected area of all spheroids between days 2 and 6. Data collected across *n* = 85 spheroids from 11 independent devices. Initial spheroid area is plotted on a log scale. Data are presented as mean ± SD. *p < 0.05

Prior to exploring tumor-vascular interactions, we sought to understand the dynamics of cancer cell growth within our model (Fig. 2). We first formed models with single cells or spheroids homogeneously distributed in the hydrogel matrix surrounding the microvessel. Tumor cell fate was determined from analysis of the fluorescence intensity of the cells over 6 days. In devices seeded with singularized cancer cells (seeded at a similar total cell density as spheroids), the fluorescence intensity in the matrix decreased slightly over time, where only a subset of single cells survived and proliferated (Fig. 2a). These cells were randomly distributed throughout the matrix with no preference for a specific location or proximity to the microvessel. In contrast, cancer cell spheroids showed robust growth over 6 days of culture (~ twofold increase in fluorescence), significantly higher growth compared to single cells (*p* = 0.011, unpaired t-test) (Fig. 2b). Cancer spheroids maintained well-defined borders with no evidence of shedding of single cells or clusters of cells. Thus, growth of cancer spheroids could be tracked by either fluorescence intensity or by spheroid area, which were strongly correlated (r^2^ = 0.677). Analyses of brain metastases from autopsy specimens previously characterized three patterns of cancer cell invasion: (1) well-demarcated growth (51%), (2) diffuse (single cell) infiltration (32%), and (3) vascular co-option (18%), where the percentages represent relative frequencies [31]. In our model, cancer spheroids matched patterns of well-demarcated growth but also displayed vascular co-option and single cell infiltration at later time points (discussed further below).

We also tested the contribution of microvessel perfusion on spheroid growth rate. The growth rate was inferred from the fold change (FC) in projected area of each spheroid between days 2 and 6 (FC_area_). In the absence of flow, spheroids displayed reduced growth compared to spheroids cultured under perfusion (*p* = 0.027, unpaired t-test) (Fig. 2c). Next, we assessed the influence of proximity to the microvessel and initial spheroid size. Spheroid growth rate was independent of vascular proximity, suggesting that nutrient transport does not lead to a gradient of spheroid growth in our model (Fig. 2d). However, we did observe a significant dependence on initial spheroid size, with the growth rate increasing exponentially with decreasing spheroid area (Fig. 2e). Cancer cells within larger tumors have less access to nutrients, suggesting that spheroids recapitulated nutrient gradients observed in patient tumors [32].

### Vascular degeneration and vessel co-option within the blood-tumor barrier

**Fig. 3:**
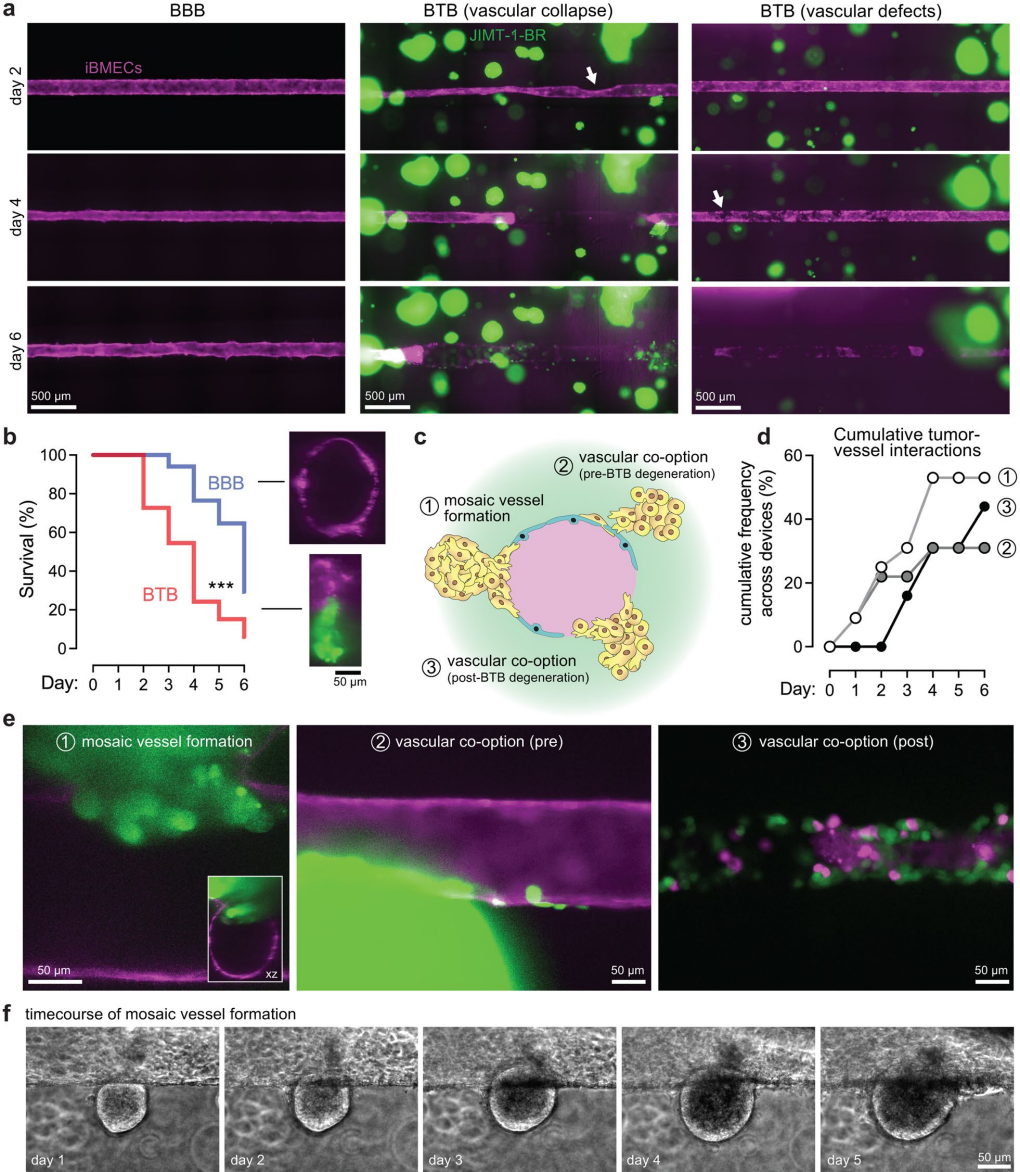
Vascular degeneration and co-option by cancer cells within in vitro metastatic blood-tumor barrier. **a** Time course images of BBB and BTB models (day 2 to day 6). Two modes of vascular degeneration (collapse and defects) were observed in the presence of metastatic spheroids. White arrows identify the locations of initial signs of vascular collapse or defect formation. iBMECs (magenta) and JIMT-1-BR (green). **b** Lifespan across *n* = 17 BBB microvessels and *n* = 32 BTB microvessels. ***p < 0.001. **c**–**d** Schematic of tumor-vessel interactions and their cumulative frequency across *n* = 32 devices. **e** Higher magnification images of tumor-vessel interactions. The inset shows the xz projection of the confocal image, while other the images are epifluorescence. Images have brightness and contrast enhanced to enable visualization of the interactions. **f** Time course imaging of perivascular tumor growth and mosaic vessel formation. See also Additional file 1: Fig. S1

Preclinical studies have shown that the BTB displays heterogeneous leakiness and hence therapeutic doses of anti-cancer agents are achieved inconsistently in most metastatic lesions [30, 33]. To better understand the barrier properties of the BTB, we conducted time-course imaging of BBB and BTB microvessels over 1 week. We first describe vascular degeneration and, in the next section, tumor—vessel interactions. The presence of cancer spheroids resulted in more rapid degeneration compared to control microvessels (Fig. 3a). We observed two modes of degeneration: (1) vascular collapse, and (2) vascular defects. Vascular collapse was most common and occurred as the endothelium physically detached from the surrounding matrix, while defect formation occurred as small holes (~ 1–5 cells) in the endothelium without changes in lumen diameter (Fig. 3a). Upon the appearance of defects or endothelium collapse, these effects quickly worsened over time as evident by widespread distribution of holes and large absence of endothelial cells at day six (Fig. 3a). We quantified the lifespan of BBB and BTB microvessels as the day on which vascular degeneration was first observed: BTB microvessels displayed a lifespan of 3.50 ± 0.23 days, while BBB microvessels displayed a higher lifespan of 5.00 ± 0.31 days (significant lower survival in BTB, *p* < 0.001, Gehan-Breslow-Wilcoxon test) (Fig. 3b).

### Mechanisms of tumor – vessel interactions in the BTB

Various mechanisms of tumor – vessel interactions have been observed in different settings [2, 3, 11, 34, 35]. During metastasis, extravasated cancer cells can remain in physical contact with the abluminal vessel wall and can proliferate along the basement membrane, a process termed *vascular co-option*. Extravasated cancer cells thrive in this perivascular niche due to proximity to nutrients and autocrine factors secreted by endothelial cells, while also being resistant to anti-angiogenic therapies which are effective in primary tumors, in part, due to the leaky vasculature (known as the enhanced permeability and retention effect). In *mosaic vessel formation*, cancer cells physically displace endothelial cells in the vessel wall, a process that can mediate intravasation of single cancer cells or cancer cell clusters. The growth of tumor spheroids can also locally compress the lumen of microvessels, resulting in *vessel constriction* and formation of dead-ends or string vessels. Finally, release of growth factors can attract endothelial cells to a nearby spheroid, a process termed *vessel pull*.

In our BTB model we observed only two tumor – vessel interactions: *mosaic vessel formation* and *vascular co-option*; we further subdivided vascular co-option based on whether cell migration occurred before or after BTB degeneration (Fig. 3c). Mosaic vessels formed in 53% of devices and roughly linearly increased in frequency over the initial 4 days of culture. Vascular co-option at early timepoints was directly associated with mosaic vessel formation (prior to BTB degeneration), while vascular co-option at late timepoints was directly associated with BTB degeneration (Fig. 3d). Using confocal microscopy, we confirmed that mosaic vessels were formed as evident by vascular cross-sections with cancer cells replacing endothelial cells in the vessel lumen (Fig. 3e). Migration along microvessels before and after BTB degeneration, suggests that vascular co-option can occur throughout progression of metastatic growth (Fig. 3e). Critically, directed proliferation and growth along blood vessels has been observed in autopsy specimens of brain metastases from solid cancers [31], but the spatiotemporal dynamics of this process are not well characterized. While multiphoton microscopy of brain metastasis in mice has visualized the early stages of tumor-vessel interactions when cancer cells are in close contact with blood vessels and perivascular growth occurs by vascular co-option [35], our studies suggest that cancer growth can also promote vascular degeneration. Spheroids in close proximity to microvessels displayed sustained perivascular growth and mosaic vessel formation through progressive displacement of endothelial cells (Fig. 3f). For a subset of devices, we continued perfusion for 2 weeks to determine terminal tumor-vessel interactions. We observed that mosaic vessels were formed in 100% of devices (*n* = 4) and that cancer cells, in some cases, completely replaced the endothelium (Additional file 1: Fig. S1).

### Time course of BTB barrier function

Brain metastases are typically identified using gadolinium-enhanced MRI; however, despite gadolinium permeation into these tumors, evidence suggests that the metastatic BTB is heterogeneously permeable to drugs and other compounds [3]. In pre-clinical animal studies, the distribution of many chemotherapeutics is similar to fluorescently-labeled dextrans, indicating a paracellular pathway for drug transport across the BTB [3, 30, 33]. To quantify how barrier properties change in the presence of metastatic cancer cells, we measured permeability of BBB and BTB microvessels by co-perfusion with non-human-specific immunoglobulin G (IgG; Cascade blue-conjugated) and anti-HER2 IgG (Alex Flour-647-conjugated). Anti-HER2 IgG is a research grade biosimilar of Trastuzumab (Herceptin®), which is widely used for treatment of human epidermal growth factor receptor 2 (HER2)–positive metastatic breast cancer [36].

**Fig. 4:**
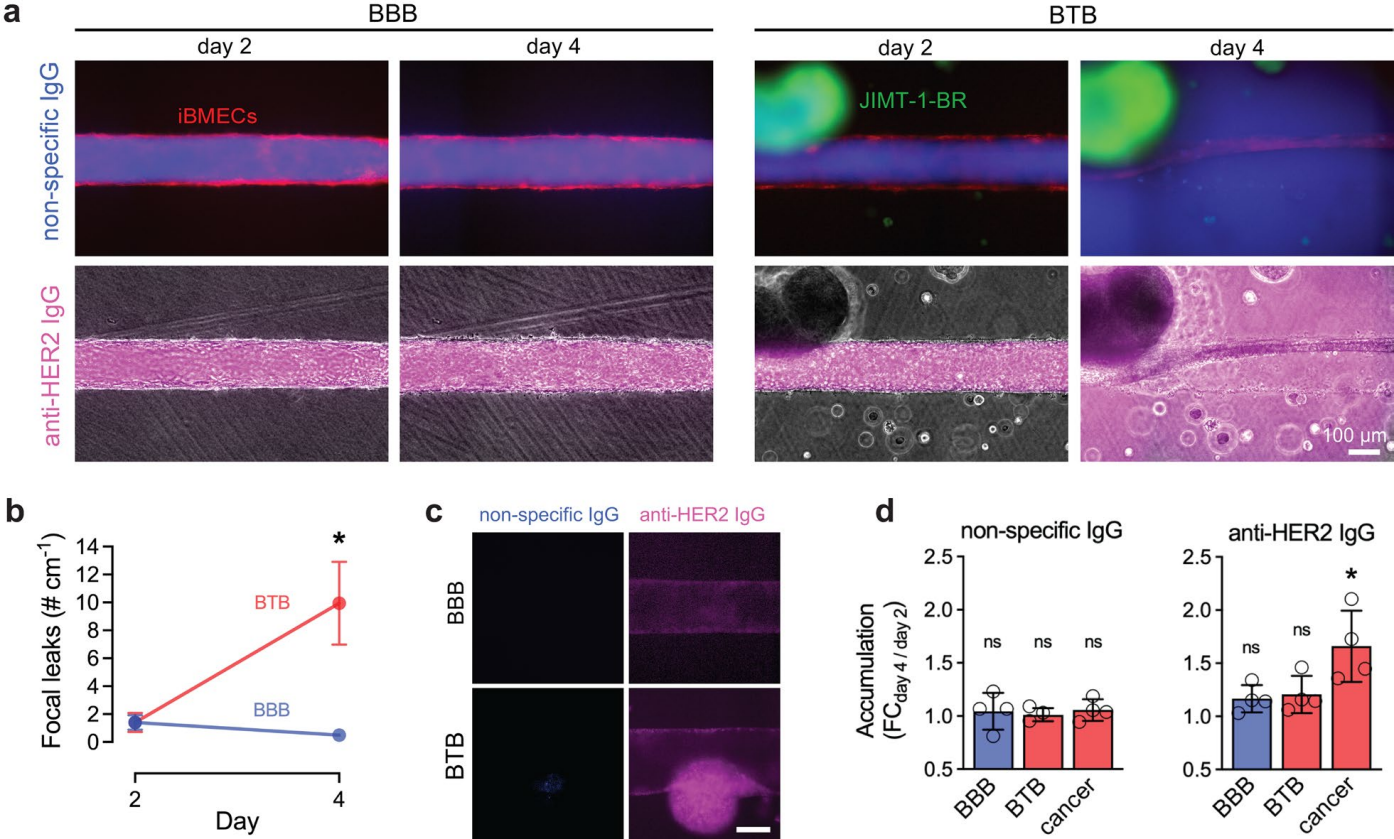
Antibody permeability and accumulation dynamics within an in vitro metastatic blood-tumor barrier model. **a** Representative images of antibody permeability with and without cancer cells. Images are at 30 min after perfusion with non-specific or anti-HER2 IgG in BBB and BTB microvessels. iBMECs (red), non-specific IgG (blue), anti-HER2 IgG (magenta), JIMT-1-BR (green). **b** Quantification of focal leaks between BBB and BTB microvessels over time (*n* = 4–5 independent microvessels per condition). **c** Representative images of antibody accumulation within the BBB and BTB at day 4. Images are normalized to day 2 fluorescence. **d** Quantification of antibody accumulation across BBB endothelium, BTB endothelium, and cancer spheroids (*n* = 4 independent microvessels per condition). Data are presented as mean ± SD. *p < 0.05. See also Additional file 1: Fig. S2

From time-lapse fluorescence images, we found that both BBB microvessels and BTB microvessels displayed negligible permeability to antibody at early time points (day 2) (Fig. 4a). However, after 4 days of culture, BTB microvessels were uniquely leaky to antibodies, matching observations of physical degeneration and formation of defects in the endothelium. While limited sites of leakage were observed at day 2 for both model types and day 4 for BBB microvessels, ~ 10 leakage sites per cm were observed in BTB microvessels, representing a significant increase compared to controls (*p* = 0.027, unpaired t-test) (Fig. 4b, Additional file 1: Fig. S2a).

To probe pathways for transport, we measured the accumulation of the two compounds in the endothelium and cancer spheroids (Fig. 4c, d). Following 30-min perfusion with IgG or anti-HER2 IgG, microvessels were perfused in the absence of antibody for 2 days prior to reassessing permeability. This washout period enabled us to determine how antibodies accumulated across conditions and locations. Critically, these experiments were conducted prior to degeneration of BTB or BBB microvessels, so that accumulation in cancer cells or spheroids, or endothelial cells, resulted from transport at day 2 of initial exposure to the IgGs. We found that non-specific IgG did not accumulate in endothelial cells or cancer cells (*p* > 0.05 for all compartments as tested by a one sample t-test to a hypothetical value of 1.0); however, anti-HER2 IgG was significantly accumulated in cancer spheroids (*p* = 0.029), without significant accumulation in the endothelium of BBB or BTB microvessels (*p* = 0.081 and 0.099) (Fig. 4c, d). HER2 antibody–drug conjugates (ADCs, 198 kDa) are able to cross the BTB in vivo but not 3 kDa dextran [37]. In vitro studies have suggested that uptake is limited to a subset of endothelial cells that support an endocytic transcellular pathway for transport [37]. Our results corroborate these findings, suggesting that Herceptin but not dextran can accumulate in the endothelium of the BTB and subsequently accumulate within perivascular metastatic tumors, even prior to paracellular barrier breakdown which is highly heterogeneous within metastatic lesions in vivo. Similar experiments were conducted using fluorescently-labeled 3 kDa dextran and fluorescently-labeled bovine serum albumin (BSA) (data not shown); paracellular focal leaks were also increased in BTB models at day 4 for these two solutes. Albumin accumulated in both cell types (endothelial and cancer), without significant differences between BBB and BTB microvessels, while 3 kDa dextran displayed no intracellular accumulation.

### Metastatic BTB displays unique gene expression

The full repertoire of tumor-vessel interactions at the BTB is not well understood as most studies focus on functional assays such as permeability. To characterize changes in the BTB with genome-wide resolution, we performed transcriptomic profiling of iBMECs within BBB and BTB microvessels using bulk RNA sequencing. A day two timepoint was chosen to identify changes in BTB phenotype prior to BTB degeneration, mosaic vessel formation, and vascular co-option. This approach minimized cancer cell contamination as we lysed endothelial cells in microvessels that did not display mosaic vessels.

**Fig. 5:**
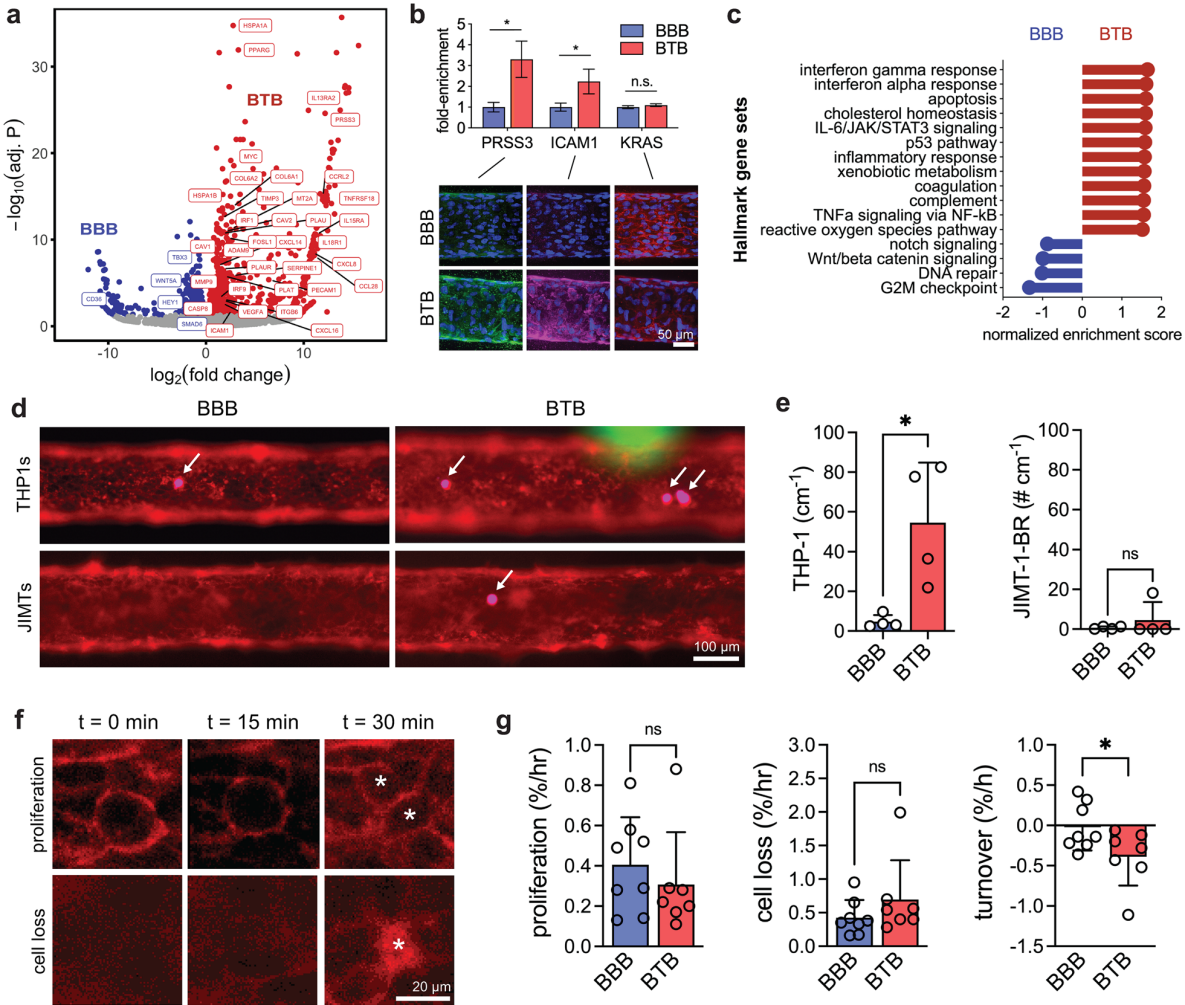
Blood-tumor barrier phenotype within in vitro metastatic lesions. **a** Volcano plots depicting significantly (adjusted *p* < 0.05) upregulated genes (blue) and downregulated genes (red) between BBB and BTB microvessels. Bulk RNA was collected from control microvessels (*n* = 3) and microvessels surrounded by JIMT-1-BR spheroids (*n* = 3), two days after seeding of iBMECs. **b** Semi-quantitative validation of PRSS3, ICAM-1, and k-ras protein levels (*n* = 4). Representative immunofluorescence images of BBB and BTB microvessels at day 2 are shown with DAPI-labeled nuclei in blue. **c** Lollipop plot of select Hallmark gene sets enriched and depleted in BTB microvessels. **d**–**e** Representative images and quantification of THP1 (monocyte-like) immune cell and JIMT-1-BR cancer cell adhesion to BBB and BTB microvessels (*n* = 4). Arrows denote adherent cells. iBMECs (red), JIMT-1-BRs (green), THP-1 s (magenta). **f**–**g** Representative images and quantification of cell turnover events (proliferation and cell loss) between BBB and BTB microvessels (*n* = 7–8). Asterisks denote proliferation and cell loss events. Turnover is calculated as the difference between rates of cell proliferation and cell loss. Data are presented as mean ± SD. Statistical analysis was performed using a student’s unpaired t-test (two-tailed with unequal variance); *p < 0.05. See also Additional file 1: Figs. S3, S4 and Additional file 2: Data S1

We observed distinct clustering of BBB and BTB endothelium using principal component analysis (PCA) (Additional file 1: Fig. S3a) and identified 988 BTB-enriched and 480 BBB-enriched transcripts (Fig. 5a). To validate these findings, we conducted semi-quantitative immunofluorescence for three proteins: serine protease 3 (*PRSS3*), intracellular adhesion molecule 1 (*ICAM1*), and k-ras (*KRAS*). BTB enrichment was confirmed at the protein level for serine protease 3 (*p* = 0.022, unpaired one-tailed t-test) and intracellular adhesion molecule 1 (*p* = 0.048), while a non-differentially expressed transcript (*KRAS*) maintained similar protein expression between both models (*p* = 0.187) (Fig. 5b, Additional file 1: Fig. S2c). To predict phenotypic differences between the BBB and BTB, we conducted gene-set enrichment analysis (GSEA) on Hallmark gene sets (Fig. 5c, Additional file 1: Fig. S4). BTB-associated Hallmark gene sets and transcripts driving their enrichment included: interferon alpha/gamma responses (*IRF1*, *IRF5*, *CCL2*, *CCRL2*, *ICAM1*), apoptosis (*CASP8*, *MMP2*, *TNFRSF21*), IL-6 signaling (*IL6*, *IL18R1*, *TNFRSF1A*, *CXCL10*), coagulation (*TIMP3*, *PECAM1*, *MMP9*), complement (*PRSS3*, *PLAT*, *PLAUR*, *SERPINE1*, *ADAM9*), hypoxia (*VEGFA*, *FOS*, *FOSL2*), among many others. BBB associated Hallmark gene sets included those associated with canonical BBB functions including wnt-beta catenin signaling and NOTCH signaling (*WNT5A*, *HEY1*).

Canonical endothelial transcripts (i.e. *KDR*, *CDH5*, *VWF*) were not uniformly altered within the BTB endothelium, suggesting that endothelial identity was not perturbed by cancer co-culture (Additional file 1: Fig. S3b). Given recent findings on iBMEC expression of epithelial transcripts [38], we sought to determine if cancer co-culture mediates vascular changes by augmenting epithelial identity of iBMECs. However, we found that most epithelial transcripts (i.e. *CDH1*, *EPCAM*, *CLDN6*) were not differentially expressed between the two models. Additionally, breast cancer marker genes were not broadly upregulated in iBMECs in the BTB model, suggesting minimal contamination of non-endothelial cells (Additional file 1: Fig. S3b); cancer associated transcripts that were upregulated including *ESR1* and *PLAU* are known to be expressed by endothelial cells [39, 40]. Lastly, we benchmarked our findings to recent transcriptomic profiling of endothelial cells isolated from normal brain tissue and from patients with lung adenocarcinoma metastases [41]. 10% of BTB-enriched genes found here were also upregulated in endothelial cells isolated from lung adenocarcinoma metastases, including transcripts involved in epithelial-to-mesenchymal transition (*SERPINE1*), angiogenesis (*VEGFA*, *CNN1*) and extracellular matrix organization (*ITGB6*, *ITGA5*, *COL6A1*, *COL6A2*) (Additional file 2: Data S1). Since endothelial isolations from human tissue also contain contaminating mural and glia cells, a more precise benchmarking of our findings is not possible.

### Metastatic BTB displays elevated immune cell adhesion and endothelial turnover

Gene set enrichment analysis was suggestive of diverse functional differences within the BTB, as well as loss of canonical BBB functions. We explored various functional responses between BTB and BBB microvessels, including adhesion of cancer/immune cells and endothelial turnover. Post-capillary venules are the preferential site of cancer cell and immune cell extravasation due to low shear stress and unique protein/gene expression [42]. To probe differences in cell adhesion to the endothelium, we perfused BTB and BBB microvessels with fluorescently labeled cancer cells and monocytes (Fig. 5d). The BTB displayed significantly increased adhesion of monocytic cells to the endothelium (~ 55 cm^−1^) compared to BBB microvessels (~ 5 cm^−1^) (*p* = 0.017, unpaired t-test) (Fig. 5e). As monocyte-derived macrophages accumulate in brain metastases [43], our model recapitulates the early stages of the transmigration cascade. This same effect was not observed following perfusion with single cancer cells (*p* = 0.421), suggesting differences in the mechanism of adhesion. Although adhesion of cancer cells to the endothelium were rare events in both models, these cells remained adherent for multiple days validating this model for studies of early stages in the metastatic cascade. In mouse models of brain metastases, the BTB endothelium expresses tumor necrosis factor (TNF) receptors conferring selective vulnerability to TNF-induced permeabilization compared to the BBB [44]. Indeed, multiple TNF receptors were upregulated in BTB microvessels, including *TNFRSF1A*, *TNFRSF4*, *TNFRSF9*, *TNFRSF18*, TNFRSF21. Furthermore, monocyte-derived macrophages (MDMs) are enriched in human brain metastases [43, 45], consistent with the BTB being conducive of monocytic infiltration. Immune cell adhesion is likely mediated by an upregulation of both endothelial surface adhesion molecules (i.e. *ICAM1*) and TNF receptor superfamily members.

To determine the influence of tumor spheroids on endothelial cell dynamics, we quantified iBMEC proliferation and cell loss prior to vascular degeneration (at day 2). Cell proliferation and cell loss events were manually counted from time-lapse phase contrast microscopy focused on the microvessel midplane and are reported as the number of events per hour (% h^−1^) (Fig. 5f). In control BBB microvessels, the rates of cell proliferation, cell loss, and overall turnover were similar to previous measurements [18]. However, BTB microvessels displayed slightly lower rates of cell proliferation (*p* = 0.458, unpaired t-test) and slightly higher rates of cell loss (*p* = 0.260), leading to significantly lower cell turnover (*p* = 0.047) (Fig. 5g). The negative turnover shows that cell loss dominates in the presence of metastatic spheroids, matching observations of vascular degeneration at late time-points. Cancer cells can mediate endothelial damage and angiogenesis by the secretion of soluble factors [46, 47] and by direct tumor-vessel interactions [48]. Indeed, BTB-enriched transcripts included mediators of apoptosis (*CASP8*) that are induced by cancer secreted factors and death receptor 6 (*TNFRSF18*) a master regulator of tumor cell-induced endothelial necroptosis [48]. iBMECs show angiogenic activity in the presence of growth factors [49] and VEGF signaling was upregulated at the transcriptional level; however, we did not observe angiogenic sprouting within the BTB model, which was also not observed in our prior model of metastatic breast cancer [11]. These observations are likely model dependent and cancer cell type dependent, as observed in multiphoton imaging studies of the metastatic cascade in vivo [35].

### Mechanisms of BTB degeneration and dysfunction

BTB phenotype is derived from both physical and chemical interactions between cancer cells and the brain endothelium. As elevated immune cell adhesion and reduced endothelial cell turnover were observed at early time points prior to vascular degeneration and direct cancer-endothelial contact, chemical interactions likely represent a key mediator of BTB phenotype. To explore chemical interactions further, we conducted experiments in 2D Transwell models using: (1) conditioned microvessel media, and (2) co-culture with cancer spheroids (Additional file 1: Fig. S5a). These experiments remove contributions from physical interactions by depleting conditioned media of cells (by centrifugation) and by using cancer spheroids in the basolateral chamber enabling only chemical crosstalk. Interestingly, exposing 2D iBMEC monolayers to BBB or BTB-conditioned media did not elicit changes in barrier function over 1 week (*p* = 0.910, one-way ANOVA) (Additional file 1: Fig. S5b). Similarly, direct co-culture of spheroids in the basolateral chamber of 2D Transwells did not induce barrier loss but instead resulted in a small increase in TEER values (*p* = 0.039, paired t-test) (Additional file 1: Fig. S5c). These findings suggest that 2D and 3D microenvironments may possess differences in cancer-derived factors and/or iBMEC responses to cancer-derived factors. Indeed, in 3D models, cancer spheroid proximity to endothelial cells is greatly reduced compared to Transwells (~ 2 mm in Transwells) and cancer growth is significantly elevated (~ 1.15-fold growth in Transwells to ~ twofold growth in 3D) (*p* = 0.031, unpaired t-test) (Additional file 1: Fig. S5d).

To probe chemical factors that may mediate BTB phenotype, we performed ELISA for six analytes in perfusate from BBB and BTB microvessels collected at day 2 (Additional file 1: Fig. S5e–f). We note that analyte concentrations were highly variable in BTB microvessels compared to BBB microvessels (concentration standard deviation was 13-fold higher), suggesting that differences in spheroid density, size, and proximity may alter analyte concentrations and, in turn, BTB phenotype. TNFα receptor (TNFR1), TNFα, and VEGF were not significantly altered between the two perfusates (*p* > 0.05, unpaired t-test). Only interleukin-8 (IL-8, *CXCL8*) was significantly elevated in BTB microvessel perfusate (fold change = 11; *p* = 0.013), while IL-6 displayed small but non-significant increases (fold change = 5; *p* = 0.079) and IL-1b displayed small but non-significant depletion (fold change = 2; *p* = 0.094).

### Macrophages augment BTB phenotype

Human brain metastases originating from primary breast cancer are comprised of ~ 30% immune cells, including resident microglia, monocyte-derived macrophages (MDMs), neutrophils, and T cells [45]. MDMs are particularly enriched in brain metastases and are localized to the perivascular region [43], suggesting that significant immune cell extravasation occurs during tumor progression. However, the contribution of macrophages to BTB phenotype remains unknown [3]. Tissue-engineered models are uniquely suitable for exploring immune cell contributions to tumor progression by avoiding issues of species-to-species differences in animal models [5].

**Fig. 6:**
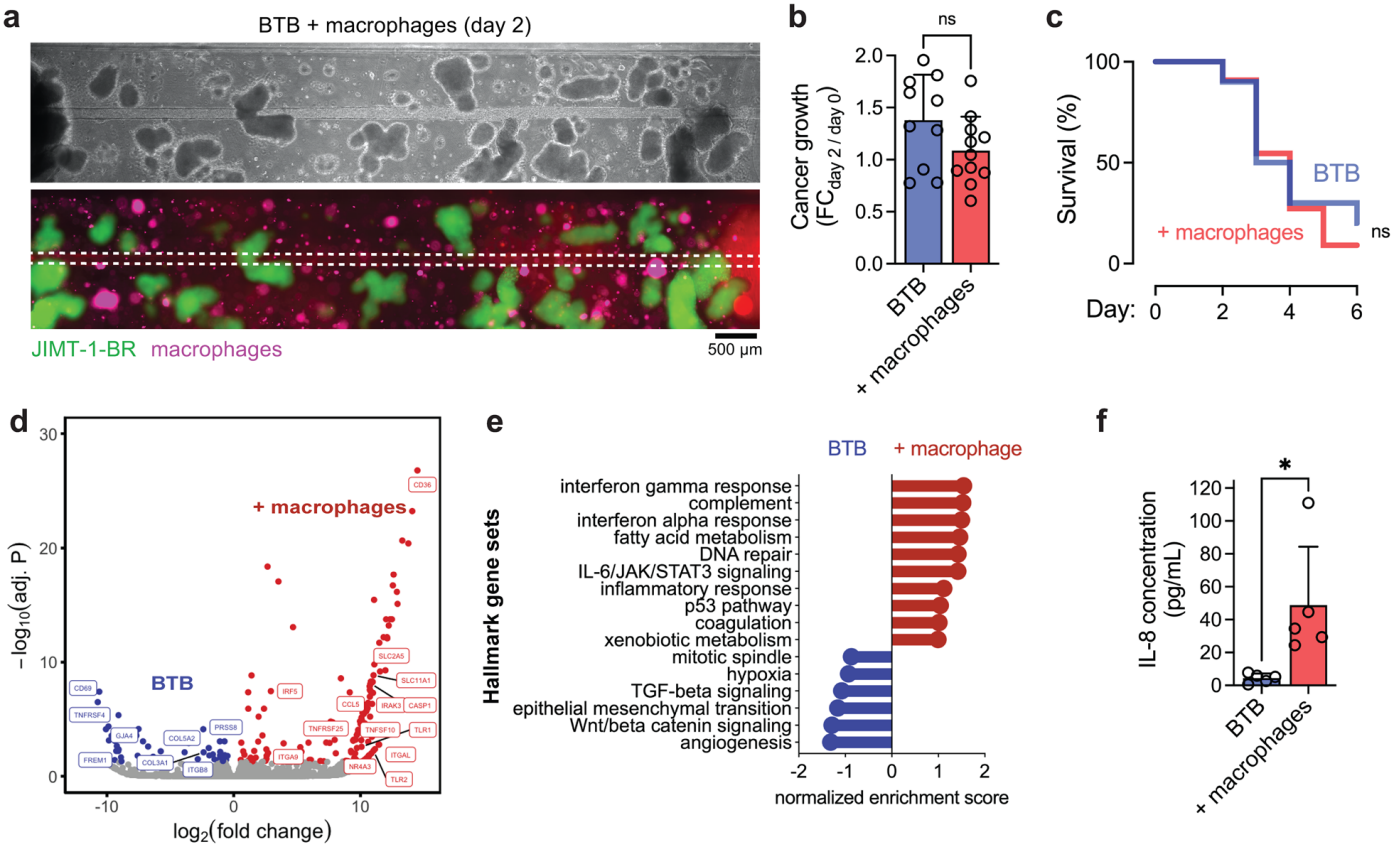
Blood-tumor barrier phenotype in the presence of macrophages. **a** Representative image of BTB model with CellTracker-labeled macrophage co-culture. iBMECs (red), JIMT-1-BR (green), macrophages (magenta). **b**–**c** Quantification of BTB spheroid growth and microvessel lifespan with and without macrophage co-culture. End points determined when iBMECs are > 50% detached or collapsed from the hydrogel. Quantification across *n* = 10 BTB devices and n = 11 BTB + macrophage devices. **d** Volcano plots depicting significantly (adjusted p < 0.05) upregulated genes (blue) and downregulated genes (red) between BBB and BTB microvessels (*n* = 3 replicates each). Bulk RNA was collected from control microvessels (*n* = 3) and microvessels surrounded by JIMT-1-BR spheroids (*n* = 3), 2 days after seeding of iBMECs. **e** Lollipop plot highlighting selected Hallmark gene sets enriched and depleted in BTB microvessels. **f** Comparison of IL-8 concentration between BTB and BTB + macrophage microvessels (*n* = 5 microvessels per condition). Data are presented as mean ± SD. *p < 0.05. See also Additional file 1: Figs. S3, S4 and Additional file 2: Data S1

To explore the contributions of monocyte-derived macrophages, we differentiated a monocytic cell line into macrophages using phorbol 12-myristate 13-acetate (PMA) and seeded these cells into the hydrogel matrix of the BTB model (Fig. 6a). Interestingly, tumor growth was slightly, but not statistically significantly, reduced by macrophage co-culture (*p* = 0.097, unpaired t-test) (Fig. 6b). Macrophage co-culture did not affect rates of microvessel degeneration (*p* = 0.913, Gehan-Breslow-Wilcoxon test) (Fig. 6c). To determine the effects of macrophage co-culture in an unbiased manner, bulk RNA sequencing of iBMECs was conducted at day 2. The magnitude of gene expression differences was much lower than comparison of BTB and BBB microvessels (Additional file 1: Fig. S3a). However, we identified 142 genes upregulated and 52 genes downregulated in the presence of macrophages (Fig. 6d). While macrophage marker genes were not broadly upregulated, increased expression of some markers suggests possible low levels of contamination (i.e. *CD68*, Additional file 1: Fig. S3b). Determining the degree of contamination is challenging as many gene families enriched in immune cells can also be expressed in endothelial cells and are responsive to inflammatory conditions. For example, toll-like receptors (TLRs) are widely expressed by endothelial cells and expression is increased in response to TLR ligands [50]; BTB microvessels with co-cultured macrophages displayed increased *TLR1* and *TLR2* expression. Transcripts depleted from iBMECs in BTB microvessels with macrophages included those involved in extracellular matrix organization (*COL17A1*, *COL3A1*, *COL5A2*). Hallmark gene sets associated with macrophages included interferon gamma responses (*CASP1*, *IRF5*, *CCL5*), among many other pathways, suggesting that macrophages can further augment BTB phenotype (Fig. 6d, e, Additional file 1: Fig. S4). To understand possible inflammatory cues that may mediate these gene expression changes, we compared analyte concentrations in microvessel perfusate with and without macrophage co-culture. IL-8, IL-1b, and TNFα were significantly elevated in BTB models with macrophage co-culture (fold changes = 11, 3.5, and 1.7, respectively) (*p* = 0.024, 0.004, and 0.045, respectively; unpaired t-test) representing possible factors secreted by macrophages or produced by cancer/endothelial cells in response to macrophage co-culture. The IL-8 concentration was ~ 11-fold higher with macrophage co-culture, representing the largest fold-change across analytes (Fig. 6f). Previous in vitro studies identified the effect of IL-8 on endothelial cells including tight junction downregulation in a dose- and time-dependent manner [51]; however, these findings are at concentrations orders of magnitude higher than levels measured here. Further studies are needed to determine functional differences induced by macrophage co-culture, but they appear to be more nuanced then directly augmenting cancer growth or microvessel degeneration.

## Conclusions

We developed a three-dimensional tissue-engineered model of the human blood-tumor barrier (BTB). During metastatic cancer, blood–brain barrier phenotype is augmented by the perivascular growth of metastatic cancer cells as well as changes in the composition of the perivascular space (e.g. infiltration of immune cells). We identified critical changes occurring within the BTB including vulnerability to vascular degeneration and defect formation, increased immune cell adhesion, mosaic vessel formation, changes in BMEC gene expression, and increased immune cell adhesion and turnover. Macrophage co-culture augmented BTB phenotype with distinct gene expression profiles of the endothelium likely mediated by elevated cytokine exposure (including IL-8).

We utilized iPSC-derived BMEC-like cells (iBMECs), which exhibit in vivo-like barrier properties and have been recently applied to study tumor-vessel interactions during triple negative breast cancer cells [52] and glioblastoma [53]. We identify distinct tumor-vessel interactions from our previously reported model of metastatic breast cancer, which utilized primary endothelial cells and breast cancer tumor spheroids grown in mouse [11]. Here, we found that the presence of metastatic breast cancer spheroids induced vascular defects including cell loss and local endothelium collapse. Accompanying these processes, we observed both mosaic vessel formation and vascular co-option events that accumulated over time. Vascular co-option was relatively rare in our system, suggesting that indirect interactions between cancer and endothelial cells also mediate phenotypic differences. Previous analysis of serum from patients with breast cancer and cerebral metastases found increased levels of CX3CL1 and CXCL13 (levels of CCL2 among other chemokines were not elevated) [54]. Additionally, recent work identified MMP-9 expression by cancer cells arrested on brain capillaries as a key mediator of capillary remodeling during brain metastasis [55]. Our ELISA of microvessel perfusate identified elevated IL-8 as potential mediator of BTB phenotype, with notably elevated levels in the presence of macrophage co-culture.

Our tumor-microvessel model recapitulates several aspects of the metastatic niche, including shear stress, cell-ECM interactions, direct tumor-vessel interactions, and cylindrical geometry. While our model matches the dimensions of large post-capillary venules, it lacks supporting cells. Future studies could incorporate astrocytes into the hydrogel and/or pericytes along the inner surface of patterned channels prior to seeding endothelial cells, as we have previously demonstrated [56, 57]. Indeed, other perivascular cells are key mediators of BTB phenotype in vivo [13, 14] and thus our model does not fully recapitulate interactions of these cell types. Additionally, given the challenges in achieving therapeutic concentrations in brain metastases, future studies could pair tissue-engineered microvessels with microdialysis-based approaches to directly measure drug concentrations in interstitial space.

Templated microvessels provide a highly complementary approach to mimic the BBB and BTB and decouple interactions of specific cell populations. We did not observe that the density of spheroids surrounding microvessels was related to the speed of BTB breakdown (data not shown), but further studies are needed to optimize the ratio of tumor cells to endothelial cells in both 2D and 3D to best mimic physiological conditions. Interestingly, we found that a 2D Transwell model of the BTB did not recapitulate barrier dysfunction observed in 3D; conditioned media also does not itself alter barrier properties nor does direct co-culture with cancer cells. Our findings highlight the complexity of interpreting 2D assays where physical tumor-vessel interactions are not recapitulated. Growth of cancer spheroids and microvessel collapse / degeneration are mediated, in part, by cell–matrix interactions. Evaluation of the extent of these interactions over time would provide further insight into cancer/microvessel interactions.

In summary, we performed timelapse imaging, functional measurements, and gene expression comparisons between iBMEC microvessels with and without co-culture of human breast metastatic cancer spheroids. We mapped physical and chemical tumor-vessel interactions that ultimately lead to degeneration of microvessels and loss of barrier properties. Additionally, we identified functional changes in the endothelium at early time points including altered cell turnover and increased propensity for immune cell adhesion (mediated by ICAM-1). Our results present new insight into tumor-vessel interactions during metastatic brain cancer and represent a system that can be further applied to test therapeutic interventions.

### Supplementary Information

**Additional file 1: Figure S1.** Long-term imaging of tumor-vessel interactions in blood-tumor barrier model. (a) Representative images of the BTB model over 14 days. At late time points, cancer cell growth resulted in contact with the glass slide at the bottom of the microfluidic device thereby obstructing imaging. (b) Representative image of complete co-option of channels with cancer cells at day 14. iBMECs (magenta) and JIMT-1-BR (green). **Figure S2.** Supplemental images of microvessel permeability and IgG accumulation. (a) Representative time course images of a permeability experiment for BBB and BTB microvessels. Dotted line shows the boundary between ECM and microvessel lumen. Arrows indicate sites of focal leaks along the length of a BTB microvessel at 20 min. (b) Day 2 and 4 fluorescence images of non-specific IgG (blue) and anti-HER2 IgG (magenta) accumulation. At baseline, Cascade blue delineates the cancer spheroids, but the signal does not accumulate over time. Over time, anti-HER2 IgG accumulates in the endothelium and spheroids, but not ECM. Representative ROIs used for quantification are shown in inset of the image. **Figure S3.** Details of RNA sequencing results. (a) Principal component analysis (PCA) of all samples. (b) Heatmap of log2FC of endothelial, epithelial, cancer, and macrophage transcripts in iBMECs. The first three heatmaps compare BTB to BBB microvessels, while the last compares BTB to BTB + macrophage microvessels. DEGs are labeled with asterisks. (c) Transcript abundance measurements of genes validated using semi-quantitative immunofluorescence (see Fig. 5b). **Figure S4.** Complete gene set enrichment analysis (GSEA) of Molecular Signatures Database (MSigDB) hallmark gene sets. (a) Normalized enrichment scores (NES) comparing BBB to BTB microvessels. (b) NES comparing BTB to BTB + macrophage microvessels. **Figure S5.** Exploring chemical microenvironmental regulation of the BTB. (a) Schematic of 2D Transwell experiments. iBMECs were cultured on a porous membrane and either exposed to microvessel-conditioned media or cancer spheroids in the basolateral chamber. Transendothelial electrical resistance (TEER) is measured daily. (b) BBB and BTB-conditioned media does not alter average TEER of iBMECs over six days of exposure (n = 4 – 5 biological replicates). (c) The presence of cancer cells in the basolateral chamber increases average the TEER of iBMECs (n = 3 biological replicates). (d) Quantification of spheroid fluorescence between 2 and 3D models. Fold change (FC) represents fluorescence on day 6 compared to day 1 (n = 3 and 6 biological replicates, respectively, where individual values represent average fold change across all spheroids in a Transwell or 3D microvessel). (e) ELISA results across six analytes (n = 5 perfusates from BBB and BTB microvessels at day 2). Dotted black lines show values obtained from fresh media not conditioned in microvessels. Data are presented as mean ± SD. * p < 0.05.

**Additional file 2: Data S1.** Summary of bulk RNA-sequencing data. (Tab 1) Differentially expressed genes (DEGs) between BTB versus BBB microvessels in vitro. (Tab 2) DEGs between BTB versus BBB microvessels from patient samples [41], including shared DEGs from Tab 1. (Tab 3) DEGs between BTB microvessels cultured with and without macrophages.

## Data Availability

Data are provided in the manuscript or Supplemental Data. The raw/processed data required to reproduce these findings are available from the corresponding author on reasonable request.
