# Supplementary material for: A tissue-engineered model of the blood-tumor barrier during metastatic breast cancer
Source: Fluids Barriers CNS. 2023 Nov 3;20:80. doi: 10.1186/s12987-023-00482-9 (PMC10623725; doi:10.1186/s12987-023-00482-9)
Supplement: Supplementary file 1 — Additional file 1: Figure S1. Long-term imaging of tumor-vessel interactions in blood-tumor barrier model. (a) Representative images of the BTB model over 14 days. At late time points, cancer cell growth resulted in contact with the glass slide at the bottom of the microfluidic device thereby obstructing imaging. (b) Representative image of complete co-option of channels with cancer cells at day 14. iBMECs (magenta) and JIMT-1-BR (green). Figure S2. Supplemental images of microvessel permeability and IgG accumulation. (a) Representative time course images of a permeability experiment for BBB and BTB microvessels. Dotted line shows the boundary between ECM and microvessel lumen. Arrows indicate sites of focal leaks along the length of a BTB microvessel at 20 min. (b) Day 2 and 4 fluorescence images of non-specific IgG (blue) and anti-HER2 IgG (magenta) accumulation. At baseline, Cascade blue delineates the cancer spheroids, but the signal does not accumulate over time. Over time, anti-HER2 IgG accumulates in the endothelium and spheroids, but not ECM. Representative ROIs used for quantification are shown in inset of the image. Figure S3. Details of RNA sequencing results. (a) Principal component analysis (PCA) of all samples. (b) Heatmap of log2FC of endothelial, epithelial, cancer, and macrophage transcripts in iBMECs. The first three heatmaps compare BTB to BBB microvessels, while the last compares BTB to BTB + macrophage microvessels. DEGs are labeled with asterisks. (c) Transcript abundance measurements of genes validated using semi-quantitative immunofluorescence (see Fig. 5b). Figure S4. Complete gene set enrichment analysis (GSEA) of Molecular Signatures Database (MSigDB) hallmark gene sets. (a) Normalized enrichment scores (NES) comparing BBB to BTB microvessels. (b) NES comparing BTB to BTB + macrophage microvessels. Figure S5. Exploring chemical microenvironmental regulation of the BTB. (a) Schematic of 2D Transwell experiments. iBMECs were cultured on a po [file 12987_2023_482_MOESM1_ESM.docx]

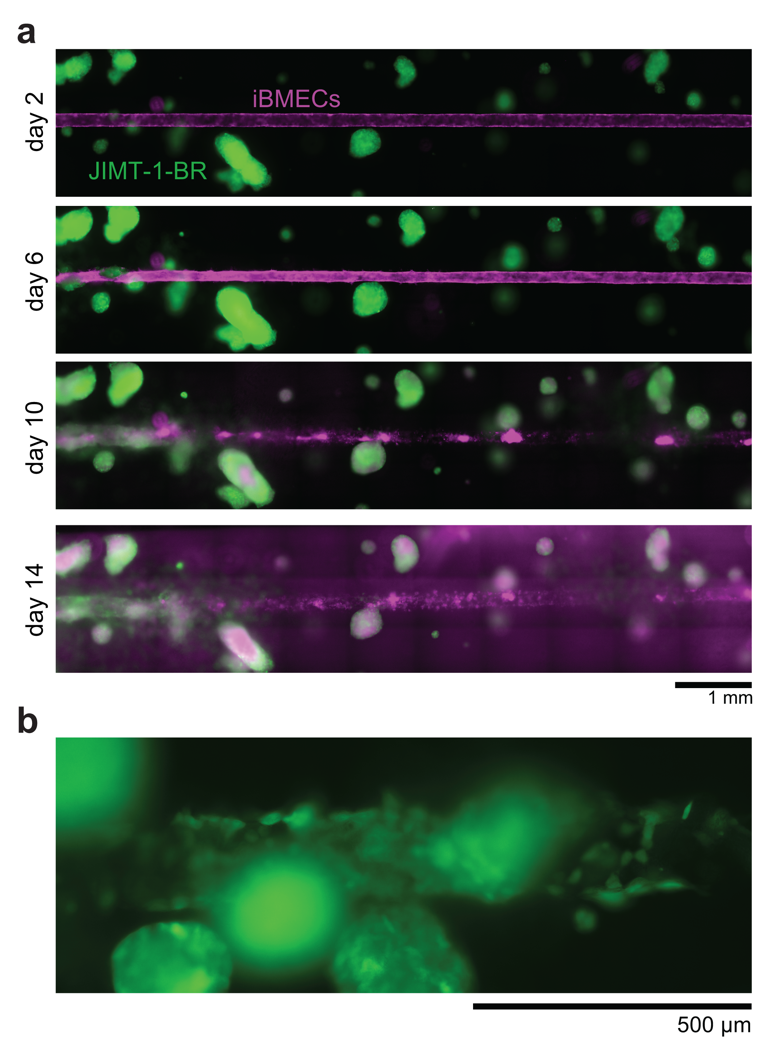


# Figure S1. Long-term imaging of tumor-vessel interactions in blood-tumor barrier model.

# (a) Representative images of the BTB model over 14 days. At late time points, cancer cell growth resulted in contact with the glass slide at the bottom of the microfluidic device thereby obstructing imaging.

# (b) Representative image of complete co-option of channels with cancer cells at day 14. iBMECs (magenta) and JIMT-1-BR (green).

**
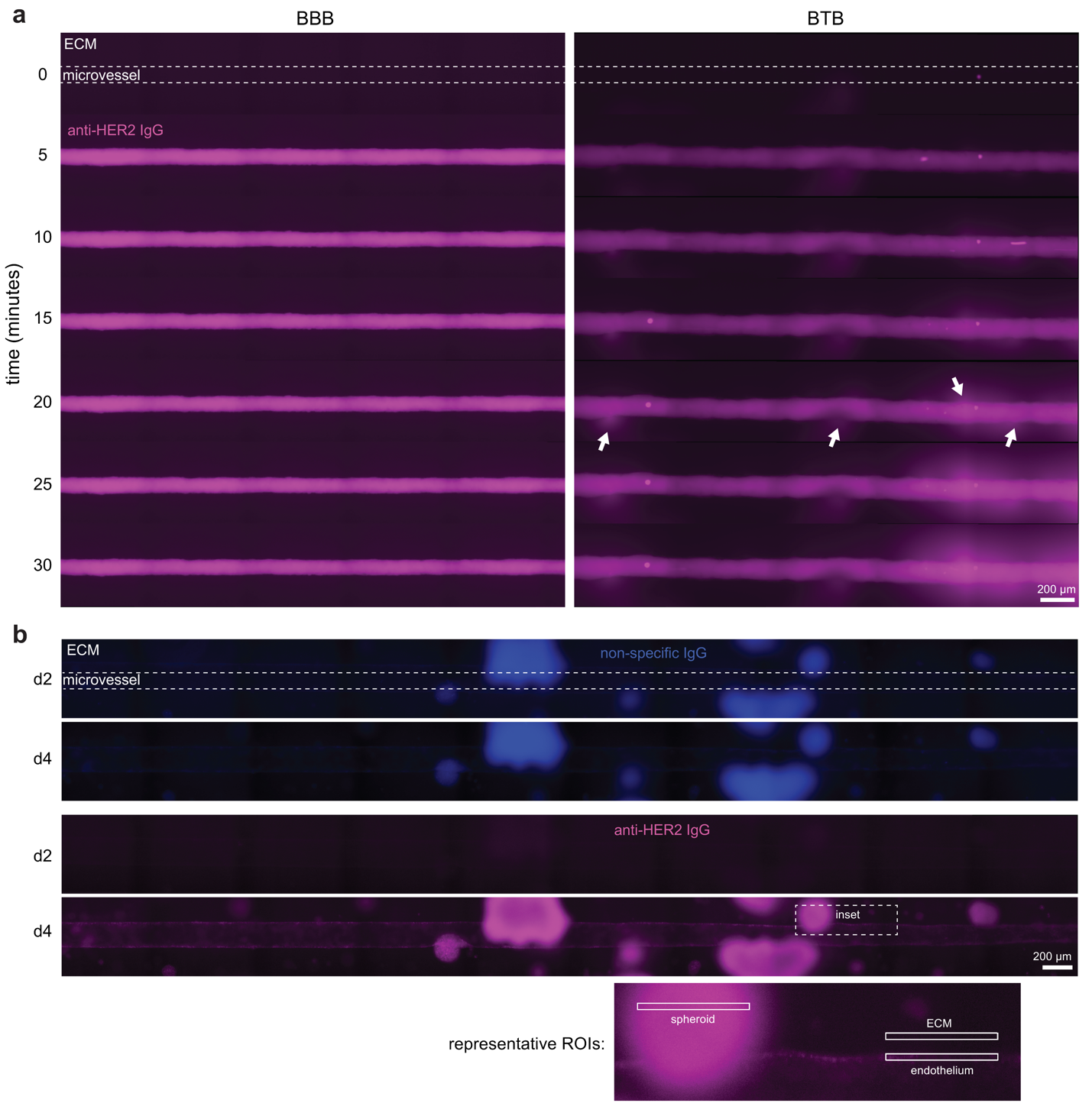
**

**Figure S2. Supplemental images of microvessel permeability and IgG accumulation.**

(a) Representative time course images of a permeability experiment for BBB and BTB microvessels. Dotted line shows the boundary between ECM and microvessel lumen. Arrows indicate sites of focal leaks along the length of a BTB microvessel at 20 minutes.

(b) Day 2 and 4 fluorescence images of non-specific IgG (blue) and anti-HER2 IgG (magenta) accumulation. At baseline, Cascade blue delineates the cancer spheroids, but the signal does not accumulate over time. Over time, anti-HER2 IgG accumulates in the endothelium and spheroids, but not ECM. Representative ROIs used for quantification are shown in inset of the image.


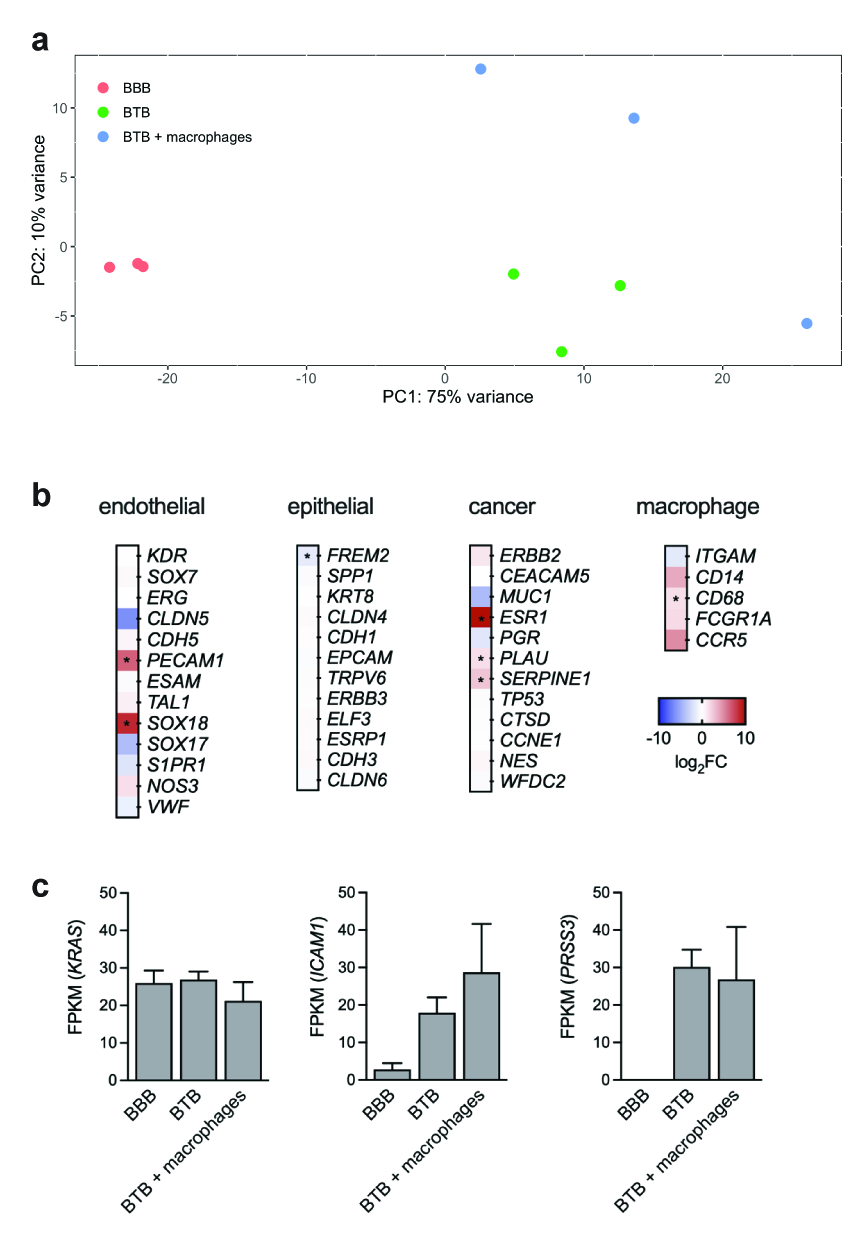


# Figure S3. Details of RNA sequencing results.

# (a) Principal component analysis (PCA) of all samples.

# (b) Heatmap of log_2_FC of endothelial, epithelial, cancer, and macrophage transcripts in iBMECs. The first three heatmaps compare BTB to BBB microvessels, while the last compares BTB to BTB + macrophage microvessels. DEGs are labeled with asterisks.

# (c) Transcript abundance measurements of genes validated using semi-quantitative immunofluorescence (see Fig. 5b).

**
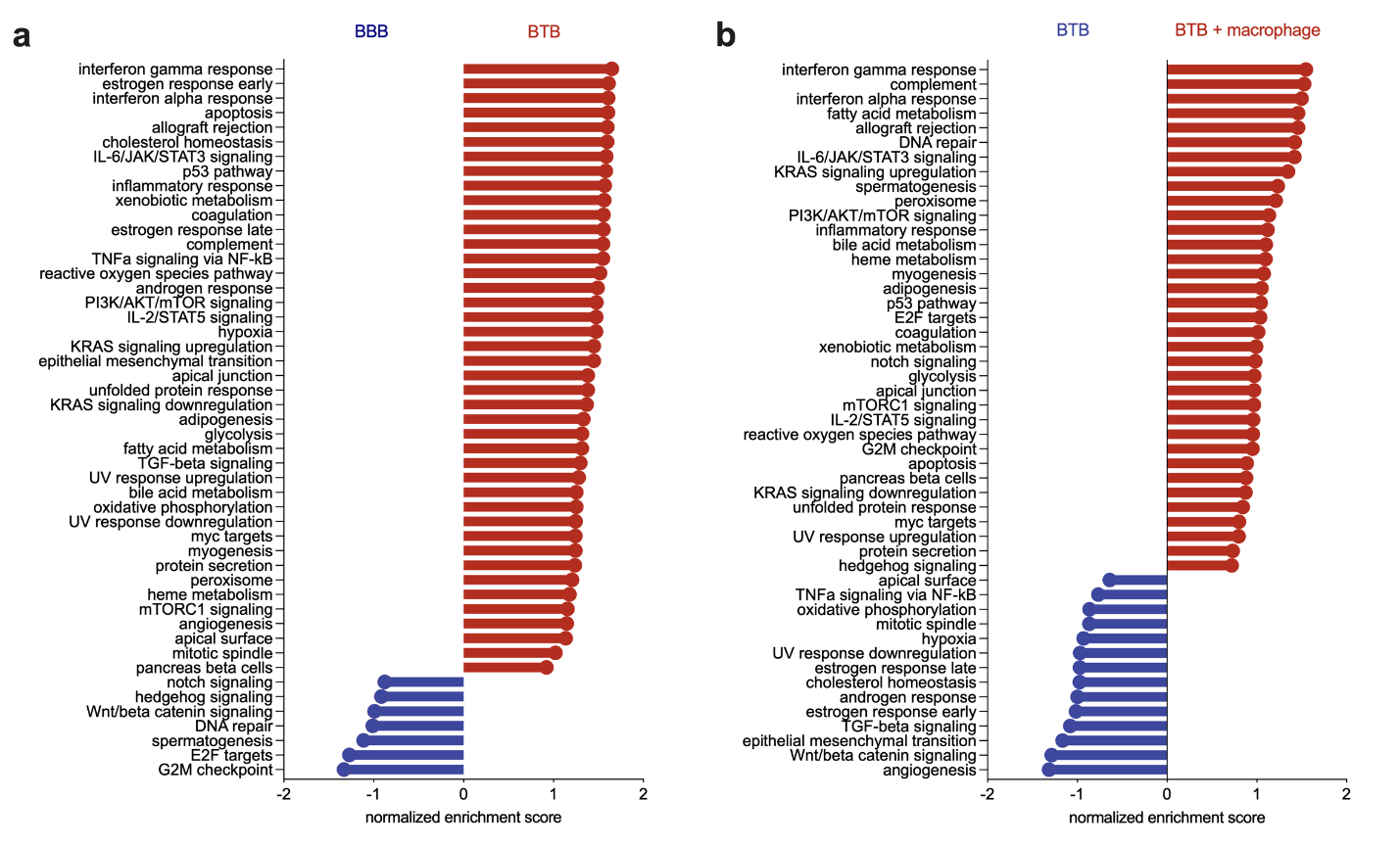
**

# Figure S4. Complete gene set enrichment analysis (GSEA) of Molecular Signatures Database (MSigDB) hallmark gene sets. (a) Normalized enrichment scores (NES) comparing BBB to BTB microvessels. (b) NES comparing BTB to BTB + macrophage microvessels.

#
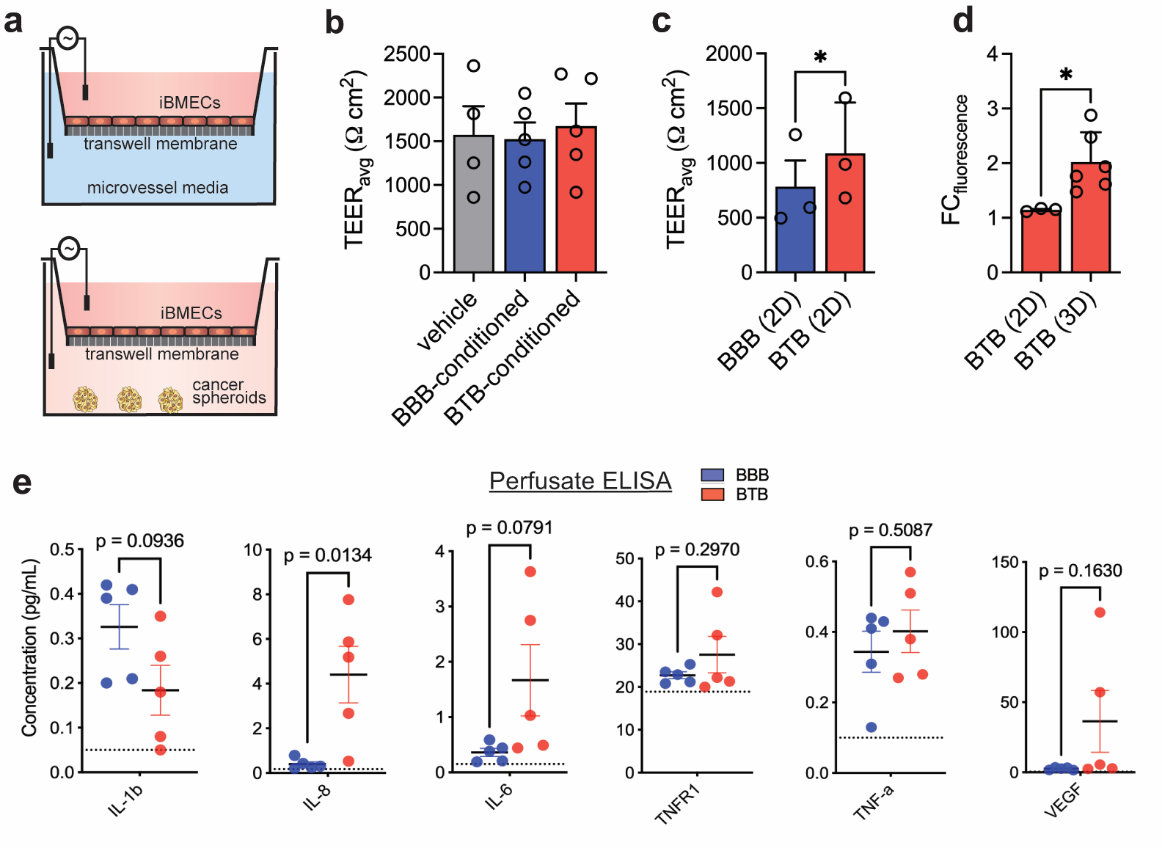


# Figure S5. Exploring chemical microenvironmental regulation of the BTB..

# (a) Schematic of 2D Transwell experiments. iBMECs were cultured on a porous membrane and either exposed to microvessel-conditioned media or cancer spheroids in the basolateral chamber. Transendothelial electrical resistance (TEER) is measured daily.

# (b) BBB and BTB-conditioned media does not alter average TEER of iBMECs over six days of exposure (*n* = 4 – 5 biological replicates).

# (c) The presence of cancer cells in the basolateral chamber increases average the TEER of iBMECs (*n* = 3 biological replicates).

# (d) Quantification of spheroid fluorescence between 2D and 3D models. Fold change (FC) represents fluorescence on day 6 compared to day 1 (*n* = 3 and 6 biological replicates, respectively, where individual values represent average fold change across all spheroids in a Transwell or 3D microvessel).

# (e) ELISA results across six analytes (*n* = 5 perfusates from BBB and BTB microvessels at day 2). Dotted black lines show values obtained from fresh media not conditioned in microvessels.

# Data are presented as mean ± SD. * *p* < 0.05.
